# Ruminal solubility and bioavailability of trace minerals in growing lambs fed varying levels of live yeast with a total mixed ration

**DOI:** 10.3389/fvets.2025.1657871

**Published:** 2025-10-08

**Authors:** Mutassim M. Abdelrahman, Ayman A. Swelum, Hani A. Ba-Awadh, Mohammed A. Al-Badwi, Mohsen M. Alobre, Gamaleldin M. Suliman, Mohammed M. Qaid, Majdi A. Bahadi, Abdullah N. Al-Owaimer

**Affiliations:** Department of Animal Production, College of Food and Agricultural Sciences, King Saud University, Riyadh, Saudi Arabia

**Keywords:** growing lambs, live yeast supplementation, total mixed ration, ruminal trace mineral solubility, systemic trace mineral bioavailability

## Abstract

**Introduction:**

This study evaluated the effects of yeast supplementation (YS) on trace mineral (TM) concentrations in blood serum, rumen fluid, and meat, as well as on the growth performance of lambs fed a total mixed ration (TMR). In addition, correlations among TM concentrations in different tissues were examined.

**Methods:**

A total of 24 healthy, growing lambs were randomly assigned to three groups (*n* = 8/group): YS0.00, YS1.50, and YS3.00 (0.00, 1.50, and 3.00 g yeast/lamb/day). Feed intake and body weight were monitored every four weeks during the 8-week trial. The samples of blood serum, rumen fluid, and meat were analyzed for Fe, Cu, Zn, I, Se, and Co using ICP-OES.

**Results and discussion:**

In the YS3.00 group, yeast supplementation significantly increased overall roughage intake. However, it decreased feed efficiency, indicating that although animals consumed more, nutrient utilization efficiency was altered. The YS1.50 group showed significantly higher serum levels of Mn, Cu, and Se (*p* < 0.05). Rumen fluid TM concentrations were significantly affected by treatment, with lower values observed in the YS1.50 and YS3.00 groups compared to the YS0.00 group. A similar pattern was observed in meat, with the YS1.50 group showing significantly higher levels of most TMs, except for I and Cu. Strong positive correlations (*p* < 0.05) were found between rumen and meat TM concentrations for Mn, Fe, Se, and I, as well as between rumen fluid and serum for Fe and Cu. In conclusion, yeast supplementation at 1.50 g/day increased trace mineral concentrations in serum and meat, with the exception of I and Cu, and improved correlations between rumen fluid and meat for Mn, Fe, Se, and I, as well as between rumen fluid and serum, in the growing lambs.

## Highlights

- The study fills a major gap in understanding how yeast supplementation (YS) affects trace mineral (TM) levels in various tissues of lambs fed a total mixed ration (TMR).
- Supplementing yeast at 1.50 g/day improved TM bioavailability, especially by increasing Mn, Cu, and Se in the blood and meat.
- Strong correlations were observed between trace mineral levels in rumen fluid, blood serum, and meat, especially for Mn, Fe, Se, and I, indicating tissue-level mineral interaction influenced by yeast.
- Yeast supplementation reduced ruminal concentrations of Mn, Fe, Zn, I, Se, and Co, suggesting enhanced systemic absorption and utilization.
- Yeast-treated lambs showed slightly better feed intake and weight gain, with no adverse effects on growth performance or health.

## 1 Introduction

In recent years, global attention to food safety and public health has increased, particularly concerning the use of antibiotics and chemical additives in livestock diets (1, 2). In addition, these additives pose potential risks, including the accumulation of chemical residues in animal products and the development of antibiotic-resistant pathogens (3). As a result, there is growing international interest in exploring natural alternatives to antibiotic feed additives for ruminants. Among these, yeast supplementation (YS) has gained prominence due to its potential benefits for both animal and human welfare (4–6).

Yeast, particularly *Saccharomyces cerevisiae*, has been widely studied for its positive effects on nutrient utilization and animal performance in small ruminants (7–11). Reported benefits include enhanced dry matter (DM) intake and body weight gain (12), modulation of volatile fatty acid profiles (13–15), stabilization of rumen pH (16, 17), and improvements in rumen microbial balance (17, 18). However, some studies have also noted reductions or no effects on growth rate and feed conversion efficiency with yeast use (19). Yeast culture has been shown to influence trace mineral (TM) absorption from the digestive tract. For instance, Jiang (20) reported higher retention of potassium (K), copper (Cu), iron (Fe), and zinc (Zn) in lambs receiving yeast culture (21). Other studies indicate that yeast supplementation can enhance concentrations of Zn, Fe, Cu, and cobalt (Co) in blood serum, rumen, liver, kidney, and meat (22, 23), suggesting its potential to improve TM bioavailability and deposition in tissues. Yeast is rich in selenium (Se); therefore, Se supplementation levels in this study were selected according to National Research Council (NRC) (24) recommendations for small ruminants (0.1–0.3 mg/kg DM), remaining well below the maximum tolerable level (MTL) of 2.00 mg/kg DM and far from toxicity thresholds (> 5.00 mg/kg DM) reported for sheep (25).

Despite these findings, the relationship between trace minerals in rumen fluid, blood serum, and meat under yeast supplementation remains unclear. Limited data exist on how different levels of yeast affect TM distribution in growing lambs, especially when fed high-fiber diets consisting of a total mixed ration (TMR). This raises an important question: Is there a significant correlation between TM concentrations in various tissues and the level of dietary yeast supplementation?

This study offers a novel evaluation of inter-compartmental mineral dynamics (rumen–serum–meat), which remain underexplored in ruminant nutrition research. The study hypothesized that live yeast supplementation enhances ruminal solubility, improves systemic bioavailability, and promotes greater tissue deposition of essential trace minerals by modulating rumen microbial activity and mineral absorption pathways. Therefore, this study aimed to evaluate how different levels of orally administered yeast affect growth performance, trace mineral metabolism, and mineral levels in the rumen fluid, blood serum, and meat of growing lambs fed a total mixed ration.

## 2 Materials and methods

### 2.1 Experimental design and lambs’ management

This study was carried out in the spring of 2020 at the Animal Experimental Station, Department of Animal Production, King Saud University, Riyadh, Saudi Arabia. The Riyadh region is characterized by a hot desert climate with extreme aridity and minimal rainfall throughout the year (26). During the experimental period, the mean ambient temperature (AT), relative humidity (RH), and temperature-humidity index (THI) were 20.0 ± 0.50 °C, 49.30 ± 1.53%, and 64.60 ± 0.58, respectively. A total of 24 healthy Awassi lambs, averaging 3.00 ± 0.50 months of age and weighing 24 ± 1 kg live body weight, were utilized in this study and randomly assigned to three treatment groups, each comprising eight individually penned lambs (one lamb per pen). The lambs were randomly allocated to three treatment groups, with eight lambs per treatment (*n* = eight replicated pens; one lamb/pen), providing adequate replication and statistical power, as supported by an *a priori* power analysis. The lambs were housed in separate 3.5 m^2^ pens to allow for natural movement and activity. Treatments consisted of a daily oral drench of 10 mL tap water (YS0.00 refers to the control group), 1.5 g live yeast dissolved in 10 mL water (YS1.50), or 3.0 g live yeast dissolved in 10 mL water (YS3.00).

All lambs were maintained under identical environmental conditions throughout the study. During the preliminary periods, each lamb was ear-tagged with a unique identification number. Subsequently, they were housed in separate pens supplied with separate feeders and water buckets. Water was provided *ad libitum*. The lambs were given two weeks to adapt to the experimental conditions before the commencement of the actual trial. They were gradually transitioned from hay to a pelleted total mixed ration (TMR) during the first week, and they were fed exclusively on the pelleted TMR during the second week.

Throughout the 60-day trial, all treatment groups followed the same feeding protocol, with the TMR offered at 3.0% of body weight in line with the NRC guidelines (24). The TMR was a balanced commercial diet formulated to meet all nutrient requirements. To see whether additional fiber influenced intake, growth, and mineral metabolism, ground Rhodes grass hay (RGH) was added at 25% above the NRC-recommended daily feed intake (DFI), alongside the TMR. Live yeast was included as a cofactor to enhance fiber digestion and mineral absorption. While the average daily TMR intake (ADTMRI) remained unchanged, the average daily RGH intake increased with yeast supplementation, suggesting improved fiber utilization and mineral dynamics. The TMR and nutritive values of the ingredients are reported in Table 1. The lambs were weighed weekly, and feed allotments (3.00% BW) were recalculated accordingly each week.

**Table 1:** Ingredients and chemical composition of the pelleted total mixed ration (TMR)^1^ diet.

Ingredients (%)	TMR	Rhodes grass hay
Barley grain	30.50	
Wheat feed	26.00	
Wheat Bran	5.00	
Palm kernel cake	17.50	
Soya Hulls	13.40	
Salt	1.00	
Limestone	2.50	
Molasses	3.00	
Acid buffer	1.00	
Commercial premix^2^	0.10	
**Total**	**100**	
Calculated analysis as dry matter basis % (DM)		
Dry matter, %	90.80	76.2
Crude protein, %	13.30	4.40
Ether extract, %	2.06	1.80
Ash, %	8.79	9.00
Nitrogen-free extract (NFE), %	53.95	29.8
Crude fiber, %	12.70	31.20
Acid detergent fiber	18.70	29.31
Neutral detergent fiber	41.40	40.54
ME, Mcal/kg	2.79	8.10
Calcium (%)	1.90	0.20
Phosphorous (%)	0.39	0.16
Iron (μg/g)	193.00	31.00
Copper (μg/g)	12.40	5.00
Zinc (μg/g)	39.30	22.00
Manganese (μg/g)	133.00	107.00
Selenium (μg/g)	0.48	0.12
Co (μg/g)	1.00	-

^1^All lambs were fed a commercially formulated pelleted TMR, which included ground Rhodes grass hay comprising 25% of the NRC-recommended daily feed intake. ^2^The commercial premix contained the following per kg of feed: 10000 IU vitamin A, 1000 IU vitamin D, 20 IU vitamin E, 300 mg Mg, 24 mg Cu, 0.6 mg Co, 1.2 mg I, 60 mg Mn, 0.3 mg Se, 60 mg Zn. Vitamin A 3335000 IU/kg, Vitamin D 335000 IU/kg, Vitamin E 16670 IU/kg, Cobalt 200 mg/kg, Copper 1,600 mg/kg, Iodine 500 mg/kg, Iron 0.0 mg/kg, Magnesium 100,000 mg/kg, Manganese 10,000 mg/kg, Selenium 100 mg/kg, Zinc 33,340 mg/kg.

At the start of the experimental period, the lambs were divided into three treatment groups (each consisting of eight lambs) according to the level of live yeast administered via oral drenching. The lambs in the three experimental groups were orally drenched once daily for 60 days using drenching guns. The treatments consisted of 10 mL of tap water containing either 0.00, 1.50, or 3.00 g of Actisaf^®^ yeast probiotic (Lesaffre, Marcq-en-Barœul, France), forming the groups YS0.00, YS1.50, and YS3.00, respectively. The inclusion levels of 1.50 g and 3.00 g/day of yeast were selected based on previous dose–response studies in lambs (27–30) and manufacturer recommendations, aiming to compare a moderate and a higher inclusion level.

### 2.2 Proximate and fiber analyses

At the end of the study, three pooled representative feed samples were randomly collected to assess their nutrient composition. The samples were oven-dried at 100 °C for 4 h to determine dry matter (DM) content and subsequently incinerated in a muffle furnace at 550 °C for 3 h to measure ash content. The AOAC (31) methods used in the proximate analyses were for ash (AOAC, 2006; 942.05), moisture (AOAC, 2006; 930.15), crude protein (AOAC, 2006; 990.03), ether extract (AOAC, 2006; 920.39), crude fiber (AOAC, 2006; 978.10), and acid (ADF) and neutral (NDF) detergent fibers (AOAC, 2006; 973.18) (32, 33).

### 2.3 Growth performance evaluation

Following a once-daily drenching of live yeast at 09:00 for 60 days to promote more stable rumen fermentation and sustained nutrient availability, the lambs were offered their TMR ration twice daily at 09:00 and 15:00 in two equal portions, as described by Mokhtar et al. (34). Average daily TMR intake (ADTMRI) and average daily Rhodes grass hay intake (ADRGHI) were determined by weighing the amount offered to each lamb, subtracting refusals using an electronic scale, and averaging the daily values over the experimental period and subsequently calculating daily feed intake (DFI). Individual TM consumption between the groups was not directly measured; feed was offered, and refusals were recorded at the group level. Therefore, intake was expressed as average daily DMI per lamb, based on group-level data.

All lambs were individually weighed once a week before feeding time. Average daily gain (ADG) for each lamb was calculated using the sum of average daily gains recorded each week. Finally, the feed conversion ratio (FCR) for each lamb was calculated as the ratio of daily feed intake to ADG. Trace mineral intake per group was not measured; only concentrations in rumen fluid, blood serum, and meat were determined to assess mineral solubility, bioavailability, and tissue deposition.

### 2.4 Preparation and extraction of the biological samples

Baseline Rumen fluid and blood serum samples were collected prior to the initiation of supplementation, and analysis confirmed no pre-treatment differences between the groups. In rumen fluid, the baseline concentrations (μg/ml) of Mn, Fe, Cu, Zn, I, Se, and Co were 50.0, 6.0, 15.0, 28.0, 1.60, 3.50, and 0.60, respectively, while in blood serum, they were 0.16, 0.60, 2.25, 0.28, 0.20, 0.15, and 0.12, respectively.

#### 2.4.1 Blood samples

In the study, blood samples were collected from a total of 24 healthy lambs at the end of the experiment. Blood serum was obtained through the initial centrifugation of the blood samples at 3000 g for 10 min at a temperature of 4 °C. The serum was immediately treated with 10% trichloroacetic acid (TCA) (1:4 Serum: TCA) and centrifuged (1,500 × g for 10 min). The supernatant was collected and stored at −20 °C until trace mineral analysis was performed on the serum.

#### 2.4.2 Rumen sampling

Rumen fluid samples were obtained from six lambs from each treated group by using an oral stomach tube connected to a vacuum pump, with the initial 50 mL discarded to minimize saliva contamination, following the protocol of Abdelrahman (22). Approximately 50 mL of rumen fluid was collected from each lamb. The samples were collected one hour before the morning feeding at the end of the experimental period. Following collection, the rumen fluid samples were poured into labeled glass tubes. Subsequently, the samples were strained through four layers of cotton gauze. Then, the strained samples were centrifuged once at 1000 g for 10 min. The resulting supernatant was transferred into Eppendorf tubes, then they were appropriately labeled and stored frozen at −20 °C for further analysis.

#### 2.4.3 Muscle sampling

At the end of the study, all lambs from the three treated groups (24 lambs) were slaughtered according to Halal practices. Carcass and non-carcass components were weighed immediately after slaughter. All carcasses were chilled at 4 °C for 24 h. *Longissimus thoracis* (LT) muscles from the 9th to 12th thoracic vertebrae on both sides were excised for analysis of meat TM concentrations.

The samples were immediately pipetted to prevent settling before removing the sample. For trace mineral analysis, 0.50 ± 0.01 g of the rumen fluid and meat samples were weighed in the digestion glass tube (in triplicate). Then, 3 mL of H_2_NO_3_ (65% Riedel-de Haen, Germany), 1–2 mL of HCl (36% Avonchem, UK), and 2 mL of H_2_O_2_ (30% w/v Avonchem, UK). The samples were digested for 30 to 60 min until the solution became clear, after which 1 mL of distilled water was added. The tubes were cooled after digestion. A 0.1 normality HCl solution was used for sample dilution to 25 mL in a flask after filtration using ashless filter papers. Subsamples were taken in tubes for analysis. The samples were stored in the refrigerator for the trace mineral analysis.

### 2.5 Minerals analysis

ICP-OES equipped with a MEINHARD nebulizer, including Type A2, was used to determine micro-mineral concentrations in the current study. Argon gas (with purity higher than 99.99%, supplied by AH Group, Dammam, Saudi Arabia) was utilized to sustain plasma and as a carrier gas. The operating conditions employed for the ICP-OES analysis were as follows: 1300.00 W RF power, 15.00 L min^−1^ plasma flow, 0.20 L min-1 auxiliary flow, 0.80 L min^−1^ nebulizer flow, and a 1.5 mL min^−1^ sample uptake rate. Axial and radial views were used for metal determination, while 2-point background correction and three replicates were used to measure the analytical signal, with the processing mode being the peak area. To ensure accuracy, emission intensities were obtained for the most sensitive lines free from spectral interference. Calibration standards were prepared by diluting the stock multi-elemental standard solution (1,000 mg L^−1^) in 0.5% (v/v) nitric acid. The calibration curves for all elements were in the range of 1.00 ng mL^−1^–1.00 μg mL^−1^ (1–1,000 ppb). ICP-OES instrument calibration was verified using standard solutions and recovery tests, with spiked recoveries ranging from 95 to 102%, confirming analytical accuracy.

### 2.6 Statistical analysis

The data collected for the current study were statistically analyzed according to a completely randomized design using the PROC MIXED procedure of the SAS software (SAS Institute, Cary, NC, version 9.4). Then, least squares means were calculated for treatment comparisons at a significance level of *α* = 0.05, using the PDIFF option of LSMEANS when *p*-values were less than 0.05 (*p* < 0.05). Pearson’s correlation coefficient was also used to interpret the relationship between trace mineral levels in blood serum, rumen fluid, and meat of the growing lambs. The sample size of eight lambs per group was considered sufficient to detect moderate treatment effects (power ≥ 0.80, *α* = 0.05), consistent with small ruminant nutrition studies. Linear and quadratic contrasts were performed to evaluate dose trends.

## 3 Results

### 3.1 Performance indicators

Table 2 illustrates the impact of yeast supplementation on various productivity variables, including daily feed intake (DFI), average daily gain (ADG), average daily Rhodes grass hay intake (ADRGHI), average daily TMR intake (ADTMRI), and the feed conversion ratio (FCR). Overall, no significant differences were observed between the treated groups across all experimental periods (Weeks 0–4, Weeks 5–8, and overall) for DFI, and similar results were observed for ADG and ADTMRI. While ADTMRI remained unchanged, average daily RGH intake increased with yeast supplementation, suggesting improved fiber utilization. It is worth noting that the lambs in the YS3.00 group exhibited a significantly higher linear increase in ADRGHI consumption compared to the other groups. However, supplementation in the YS3.00 group significantly enhanced the FCR compared to other treated groups during Weeks 5 to 8. The FCR values were similar across all groups—5.63, 5.92, and 5.20 for YS0.00, YS1.50, and YS3.00, respectively—but these differences were not significant.

**Table 2:** Effects of yeast supplementation on growth performance parameters in the growing lambs.

Variables^2^	Period	Treatments^1^ (yeast, g/lamb)			SEM	*p*-value	Contrast	
		YS0.00	YS1.500	YS3.00			Linear	Quadratic
DFI (g)	Weeks 0–4	1160.00	1205.00	1180.00	27.700	0.350	0.153	0.105
	Weeks 5–8	1656.00	1677.00	1652.00	28.100	0.790	0.239	0.190
	Overall (0–8)	1427.00	1452.00	1423.00	29.300	0.810	0.192	0.166
ADG (g)	Weeks 0–4	277.00	339.00	303.00	19.000	0.130	0.093	0.060
	Weeks 5–8	335.00	284.00	313.00	17.700	0.530	0.401	0.305
	Overall (0–8)	308.00	310.00	308.00	16.600	0.990	0.45	0.476
ADRGHI	Weeks 0–4	300.00	337.00	309.00	14.200	0.120	0.110	0.082
	Weeks 5–8	274.00^b^	303.00^a^	306.00^a^	8.980	0.020	0.024	0.051
	Overall (0–8)	286.00^b^	319.00^a^	307.00^ab^	8.290	0.010	0.025	0.059
ADTMRI	Weeks 0–4	854.00	868.00	871.00	20.050	0.790	0.702	0.679
	Weeks 5–8	1382.00	1374.00	1346.00	21.820	0.500	0.567	0.532
	Overall (0–8)	1134.00	1133.00	1116.00	20.520	0.910	0.976	0.622
FCR^3^	Weeks 0–4	4.71	4.22	4.51	0.360	0.570	0.689	0.536
	Weeks 5–8	6.41^a^	7.46^a^	5.84^b^	0.320	0.050	0.091	0.050
	Overall (0–8)	5.63	5.92	5.20	0.290	0.270	0.689	0.636

^1^Treatments: YS0.00, YS1.50, and YS3.00 represent 0.00, 1.50, and 3.00 g/animal, respectively. ^2^DFI is daily feed intake; ADG is average daily gain; ADRGHI is average daily Rhodes grass hay intake; ADTMRI is average daily TMR intake; FCR is feed conversion ratio. a and b Means within the same row with different superscripts are significantly different at a *p*-value of <0.05 due to the effect of treatment alone. ^3^FCR was calculated as the ratio of DFI to ADG.

### 3.2 Trace mineral evaluation in blood, rumen fluid, and meat

The summarized effects of varying levels of yeast supplementation (YS) on trace minerals in the blood serum of the lambs are presented in Table 3. Significant changes (*p* < 0.05) were observed in Mn, Cu, and Se concentrations, which were notably higher in the treated groups (especially in YS1.50) compared to the Y0 group. However, no significant differences (*p* < 0.05) were observed in the content of other TMs among all groups. In rumen fluid, there were significant variations (*p* > 0.05) in most of the TM concentrations (YS0.00, YS1.50, and YS3 groups per germ/head), except for I. Generally, the treated groups (YS1.50) showed significantly lower concentrations of TMs compared to the other groups. This difference was particularly pronounced in the lambs from the treated group, except for Cu and I, which showed significantly lower values in the YS1.50 group. However, Zn did not follow this trend (Table 4).

**Table 3:** Effects of yeast supplementation on trace mineral concentrations (μg/ml; Mn, Fe, Cu, Zn, I, Se, and Co) in the blood serum of the feedlot lambs.

Variables	Treatments^1^ (yeast, g/lamb)			SEM	*p*-value	Contrast	
	YS0.00	YS1.50	YS3.00			Linear	Quadratic
Mn	0.18^b^	0.19^a^	0.16^c^	0.006	0.050	0.094	0.051
Fe	0.79	0.80	0.82	0.025	0.210	0.404	0.330
Cu	3.12^b^	4.36^a^	3.12^b^	0.185	0.015	0.014	0.091
Zn	0.43	0.43	0.44	0.016	0.550	0.203	0.134
I	0.27	0.28	0.27	0.069	0.370	0.244	0.337
Se	0.18^b^	0.19^a^	0.16^c^	0.004	0.001	0.175	0.0004
Co	0.14	0.15	0.15	0.002	0.240	0.167	0.328

^1^Treatments: YS0.00, YS1.50, and YS3.00 represent 0.00, 1.50, and 3.00 g/animal, respectively. a–cMeans within the same row with different superscripts are significantly different at a *p*-value of <0.05 due to the effect of treatment alone.

**Table 4:** Effects of yeast supplementation on trace mineral concentrations (μg/ml; Mn, Fe, Cu, Zn, I, Se, and Co) in the rumen fluid of the feedlot lambs.

Variables	Treatments^1^ (yeast, g/lamb)			SEM	*p*-value	Contrast	
	YS0.00	YS1.50	YS3.00			Linear	Quadratic
Mn	46.40^a^	33.60^b^	29.00^b^	1.720	0.018	0.028	0.054
Fe	13.00^a^	7.79^b^	5.99^b^	0.630	0.004	0.011	0.021
Cu	13.30^a^	9.06^b^	9.01^b^	0.760	0.011	0.046	0.065
Zn	31.50^a^	27.50^b^	30.90^ab^	0.920	0.012	0.016	0.052
I	1.56	1.91	1.59	0.170	0.065	0.886	0.021
Se	2.18^a^	2.17^a^	1.06^b^	0.210	0.001	0.047	0.187
Co	1.48^a^	0.51^b^	0.22^b^	0.100	0.031	0.018	0.200

^1^Treatments: YS0.00, YS1.50, and YS3.00 represent 0.00, 1.50, and 3.00 g/animal, respectively. a and b Means within the same row with different superscripts are significantly different at a *p*-value of <0.05 due to the effect of treatment alone.

The results indicated that yeast supplementation at 1.50 g had a significant impact on the concentrations of most TMs in the meat of the lambs. In general, TM levels were significantly lower (*p* < 0.05) in the YS0.00 group than in the treated groups. However, no significant differences were observed in the concentrations of I among the groups (Table 5). In general, iodine, among all TMs, was not affected by YS in blood, rumen fluid, and meat.

**Table 5:** Effects of yeast supplementation on trace mineral concentrations (μg/ml; Mn, Fe, Cu, Zn, I, Se, and Co) in the meat of the growing lambs.

Variables	Treatments^1^ (yeast, g/lamb)			SEM	*p*-value	Contrast	
	YS0.00	YS1.50	YS3.00			Linear	Quadratic
Mn	16.90^a^	8.07^b^	5.79^b^	1.020	0.002	0.001	0.572
Fe	54.20^a^	47.60^b^	23.70^c^	1.430	<0.001	0.112	0.002
Cu	816.00	709.00	681.00	36.900	0.581	0.322	0.775
Zn	87.50^a^	84.10^a^	54.60^b^	2.610	<0.001	<0.0001	0.045
I	1.44	1.26	1.23	0.110	0.132	0.284	0.085
Se	35.10^a^	18.90^b^	15.70^b^	1.830	0.041	0.040	0.452
Co	1.27^a^	0.92^ab^	0.47^b^	0.150	0.0001	<0.0001	0.773

^1^Treatments: YS0.00, YS1.50, and YS3.00 represent 0.00, 1.50, and 3.00 g/animal, respectively. a–cMeans within the same row with different superscripts are significantly different at a *p*-value of <0.05 due to the effect of treatment alone.

### 3.3 Trace mineral correlation

**Figure 1:**
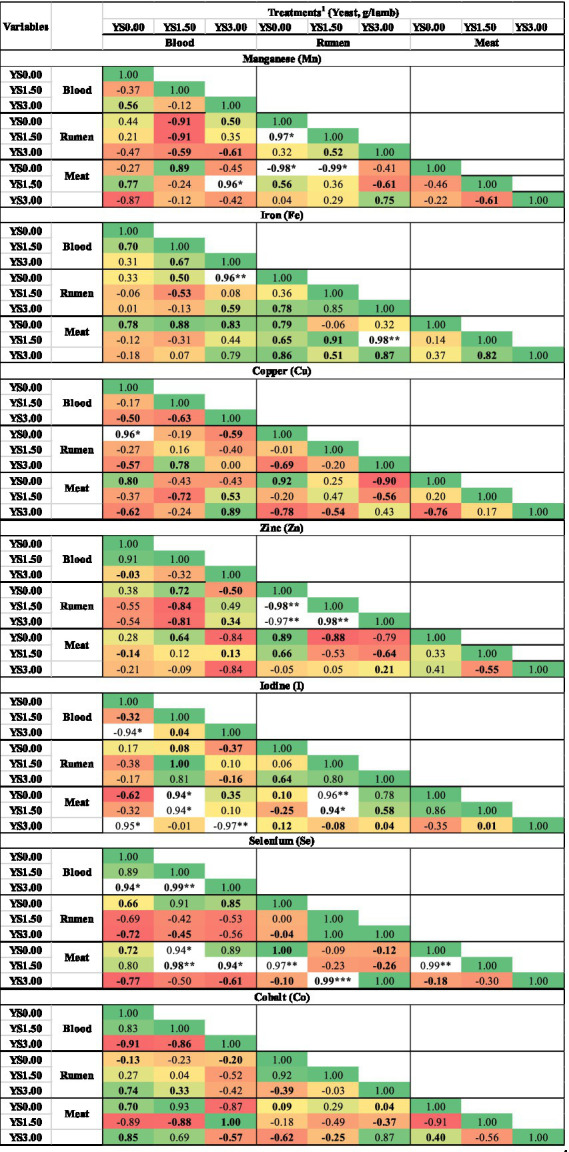
Correlation coefficient analysis of trace mineral levels between blood serum, rumen fluid, and meat samples from the growing lamb. ^1^Treatments: YS0.00, YS1.50, and YS3.00 represent 0.00, 1.50, and 3.00 g/animal, respectively. *p < 0.05; **p < 0.01; ***p < 0.001.

Correlation analysis (R^2^) was performed to understand the relationship among the supplemented trace minerals in the blood serum, rumen fluid, and meat samples. A highly significant (*p* < 0.01) correlation was observed among various studied minerals, enhancing the power of tests, and values above 0.01 are discussed here, as presented in Figure 1. The significant correlation (*p* > 0.05) between TMs and different levels of dissolved yeast in the blood serum, rumen, and meat of the growing lambs was observed for Mn, Fe, Cu, Zn, I, Se, and Co (Figure 1). In general, no significant correlations were observed between the concentrations of Zn and Co in serum and TM concentrations in the rumen fluid (Fe and Cu) and meat samples (Mn, I, and Se). However, there were notable correlations between TM concentrations in serum and those in the rumen fluid and meat samples. Specifically, the results indicated that significant correlations were observed between the concentrations of Mn, Fe, I, and Se in blood serum and their corresponding TM concentrations in the rumen fluid and meat samples. For instance, Mn in blood serum showed a strong positive correlation (R^2^ = 0.96) with Mn in rumen fluid, as well as a strong negative correlation (R^2^ = −0.97) with Mn in the meat samples. Similarly, Fe in blood serum showed a strong positive correlation (R^2^ = 0.96) with Fe in rumen fluid, and Se in blood serum showed a strong positive correlation (R^2^ = 0.94) with Se in the meat samples. Interestingly, the correlations indicate an interrelationship between TM concentrations in the blood serum, rumen fluid, and meat samples, particularly during YS administration.

During YS supplementation, Mn levels in rumen fluid showed a strong negative correlation (R^2^ ≤ 0.96) with Mn levels in meat. Fe in rumen fluid was positively correlated with Fe in meat (R^2^ ≤ 0.98; *p* > 0.05), with the effects of Fe in meat. At the highest YS level (YS1.50), Mn, Se, and I in rumen fluid demonstrated strong positive correlations with their respective concentrations in the meat samples (R^2^ ≤ 0.96 for Mn; R^2^ ≤ 0.99 for Se and I). In contrast, Zn had a negative correlation with Se in the meat samples (*p* > 0.05; R^2^ ≤ −0.88). Overall, no significant correlations were found for Cu and Co among the rumen fluid, blood serum, and meat samples.

Overall, the effect of yeast supplementation was significantly (*p* > 0.05) higher at 1.50 g on all TM concentrations in the blood serum of the growing lambs compared to the YS0.00 group. However, the concentrations of TMs in rumen fluid were lowest (*p* > 0.05) in the YS1.50 and YS3.00 groups, as expected for I concentrations, which were higher than those in the YS0.00 group. For the meat samples, TM statuses in general were lowest (*p* > 0.05) for Mn, Cu, I, Fe, Zn, Se, and Co in the YS1.50 group, except for Zn, which was significantly (*p* > 0.05) higher in the YS1.50 group.

## 4 Discussion

In the present study, the effects on overall DFI, ADG, ADCI, and the FCR were not significant. On the other hand, YS did show significant effects on ADRI in the growing lambs. Furthermore, YS supplementation with 1.50 g dissolved in tap water significantly enhanced ADCI and FCR during the 5th and 8th weeks. These findings are consistent with some previous studies (35–37) and contrast with others (38, 39).

Several studies have indicated interactions (synergism and antagonism) among trace minerals. This affects absorption, availability, utilization, and marginal deficiencies of inorganic and organic dietary components (40–42). Yeast supplementation may mitigate mineral antagonism by stabilizing rumen pH, reducing insoluble precipitate formation, and binding excess minerals to microbial cell walls, thereby facilitating improved intestinal absorption (43, 44). Dietary factors play a crucial role in the bioavailability, absorption, and accumulation of trace elements. A prime example of this is the interaction between iron and other elements. Iron exhibits antagonistic behavior with copper (Cu) and zinc (Zn). In addition, elements such as Cu, Se, and cobalt (Co) have an antagonistic interaction with molybdenum (Mo), iodine (I), zinc (Zn), iron (Fe), and magnesium (Mg). Notably, copper is particularly sensitive to inhibition by iron, Mo, and sulfur (S) (13, 45, 46). These interactions highlight the intricate relationships between dietary factors and trace element availability, further emphasizing the need to consider these dynamics in studies related to nutrition and health. In grazing sheep, an increase in Mo levels in pasture remains a significant problem, especially in the presence of dietary S. As little as 1 mg Mo per kg^−1^ of DM in pasture has a significant impact on Cu absorption and storage (42, 47). This, in turn, affects its presence in animal tissues; for example, low Cu status is linked with altered blood serum biochemical profiles, bone and nervous disorders, poor live weight gain, compromised immunity and antioxidant capacity, and impaired reproductive performance in farm animals (48, 49).

Furthermore, changes in mineral interactions may account for certain disparities. This study is consistent with previous findings (50, 51), which reported improved Se and Cu bioavailability with yeast supplementation, as well as with the study of Gowda et al. (52), who observed enhanced Cu and Zn status in lambs. However, the divergence from the findings of Abdelrahman et al. (53), who noted increases in both rumen and tissue mineral concentrations, is likely due to differences in basal diet type (forage-based vs. TMR) and mineral turnover. Excessive intake of Fe and Zn in lambs reduces Cu concentrations in the plasma and liver, possibly through the formation of insoluble mixed Fe and Cu sulfides in the gastrointestinal tract or Zn-mediated inhibition of Cu absorption (49). Therefore, high ruminal Fe might inhibit Cu absorption by precipitating insoluble sulfides, whereas excess Zn can impair Cu uptake by competing for intestinal transport sites (25, 54). In the current investigation, lower ruminal Fe and Zn levels in the YS1.50 group may have mitigated these antagonistic effects, leading to increased serum Cu concentrations. In contrast, the lower levels of Mn in meat from the YS1.50 and YS3.00 groups compared to the YS0.00 group may indicate that Mn is being prioritized for enzymatic functions in tissues with higher metabolic turnover rather than for accumulation in skeletal muscle. The YS1.50 group’s favorable associations between blood and meat concentrations of Mn, Fe, Se, and I suggest that yeast supplementation improves mineral partitioning between circulating pools and tissue storage. These relationships are consistent with the findings of Abdelrahman and Hunaiti (55), although they differ from several instances where tissue mineral concentrations did not match serum levels, presumably due to short-term supplementation or an insufficient yeast dose.

Interestingly, many minerals exhibit both synergistic and antagonistic interactions with some other minerals, such as Fe-Cu, Fe-Mn, and Zn-Mn; the balance between these minerals is more complicated and requires more care when adding them to the diets of animals (56). Therefore, in our study, we aimed to understand the relationship between Mn and different levels of dissolved yeast supplementation in the blood serum, rumen, and meat of growing lambs. Our results indicated a significant increase in serum Mn concentrations in the group receiving 1.50 g/head compared with other groups, while iodine concentrations did not differ significantly among groups. The results show a similar trend to those reported by Abdelrahman and Hunaiti (55), who found that the bioavailability of copper, zinc, and cobalt in growing lambs and their growth performance improved with supplementation of 2.00 g/day of YS. Zhang et al. (50) found that combining yeast culture with organic Se supplementation increased Se, Cu, and Zn levels in sows while also improving antioxidant capacity through increased glutathione peroxidase (GSH-Px) and superoxide dismutase (SOD) activity, ultimately leading to improved animal health and performance. The current findings are consistent with their Se findings, despite changes in species, production stage, and basal diet composition. The convergence in Se response supports the concept that organic Se from yeast (mostly selenomethionine) is more efficiently absorbed into animal tissues than inorganic sources, regardless of ruminant or monogastric physiology. Unlike Zhang et al. (50), the current investigation found declines in specific meat mineral concentrations (Fe and Mn at higher YS doses), which could reflect a mineral redistribution toward metabolic processes or antioxidant systems rather than storage in muscle tissue.

Based on the nutrient requirements of small ruminants, the National Research Council (NRC) (24) recommends an Se supplementation level of 0.10–0.30 mg/kg of diet dry matter for sheep to meet their maintenance and growth requirements. The NRC (24) and Suttle (25) indicate that the MTL of Se in sheep diets is 2.00 mg/kg DM. Chronic selenosis (alkali disease) can occur when dietary Se intake exceeds 5.00 mg/kg DM over extended periods, and acute toxicity (blind staggers) may occur above 10.00 mg/kg DM. Therefore, the Se supplementation levels used in this study were kept below the MTL of 2.00 mg/kg DM and well below toxicity thresholds (>5.00 mg/kg DM). Although the experiment was conducted in modest thermal settings during the spring, when the average THI was 64.60, which is lower than the usual heat stress (HS) level for sheep (THI ≥ 72.00), the pattern of Se enrichment in serum and meat was consistent with findings from experiments conducted under higher heat loads (50). This suggests that the antioxidant effect of Se yeast is not limited to mitigating HS. In hotter regions (THI ≥ 72), Se response increases due to oxidative stress, but the direction of change stays consistent. Studies indicate that a THI of less than 72.00 reflects no HS, values between 72.00 and 78.00 indicate mild HS, values from 78 to less than 90.00 indicate moderate HS, and values of 90.00 or higher indicate severe HS (57, 58). Under these modest thermal settings, the oxidative damage caused by heat load was most likely minimal. Nonetheless, selenium-enriched yeast may improve antioxidant defense because organic selenium from yeast (mostly selenomethionine) is easily absorbed into antioxidant enzymes such as glutathione peroxidase. This could explain the continuous increase in serum and meat Se concentrations in the YS1.50 group, despite the lack of significant ambient HS. Higher THI conditions, as found in summer trials, may make the antioxidant effects of Se yeast even more obvious due to increased reactive oxygen species production.

The current study found that yeast supplementation at 1.50 g/day (YS1.50) significantly increased serum concentrations of Mn, Cu, and Se in the growing lambs while lowering most TM concentrations in rumen fluid. This pattern is consistent with the findings of Abdelrahman and Hunaiti (55), who observed increased serum and tissue Zn, Mn, and Co levels in Awassi lambs supplemented with 2.00 g/day of yeast. These changes were accompanied by indications of improved mineral bioavailability through improved absorption efficiency, as well as improved growth performance. The reduction in rumen mineral concentrations here suggests more rapid transfer to systemic circulation, similar to the findings of Abdelrahman (22), where direct-fed microbials enhanced early mineral status in lambs. In contrast, Pal et al. (23) observed that *Saccharomyces cerevisiae* yeast supplementation, with or without TMs, increased rumen and tissue mineral concentrations in Black Bengal kids. This variation can be attributable to changes in diet type (forage-based *vs.* pelleted TMR), mineral background levels, and yeast form and dose. In the current study, the reduction in rumen mineral concentrations at the YS1.50 and YS3.00 levels may reflect improved absorption efficiency (minerals being transferred more quickly from the rumen to the bloodstream), whereas the higher rumen mineral concentrations reported by Pal et al. (23) may indicate slower turnover rates or higher rumen binding capacity under their feeding conditions. In all experiments, yeast supplementation appeared to boost systemic mineral consumption, most likely through improved rumen microbial activity and the stabilization of ruminal pH, which enhances the solubility and transport of certain trace elements.

However, the meat samples from the lambs that were fed diets containing YS showed significantly lower levels, including an overall mean of Mn, Cu, I, Fe, Se, Zn, and Co in general (YS1.50 and YS3.00). Most of the TMs were lower in the YS1.50 group compared to the YS0.00 group. Our data showed a decrease in TM levels in rumen fluid, followed by an increase in blood serum and a reduction in the meat samples. Reducing TM in rumen fluid influenced TM deposition in meat. A stronger positive correlation of Mn and Fe among blood, rumen fluid, and meat samples was observed at the highest YS supplementation compared with the control (YS0.00). The concentrations of I and Se in blood serum were significantly highly correlated with their levels in the meat samples, especially at the YS1.50 level. These results indicated that there was a relationship between the concentrations of TM in the blood and their corresponding levels in the meat samples. The positive correlations suggested that the levels of Mn, Fe, Se, and I in the blood were reflective of their concentrations in the meat samples (25, 56, 59), with YS1.50 showing a particularly strong association. On the other hand, there was a negative correlation between Mn and Zn in rumen fluid and Mn and Zn in the meat samples; this reinforces that some minerals have both synergistic and antagonistic effects on some other minerals (56). In this study, the correlation results indicated that the manipulation of rumen digestibility through YS at 1.50 g had significant effects on TMs in the blood samples and, consequently, manifested as discernible alterations in TM levels in the meat samples.

Differences between studies may also reflect the mineral composition of the base diet. According to previous studies (25, 54), high dietary Fe, S, or Mo levels can reduce Cu and Se absorption. In this investigation, the reduction in rumen Fe and Zn at the YS1.50 level may have relieved these antagonistic effects, contributing to the observed serum Cu increase. Pal et al. (23) did not report such reductions, presumably due to lower dietary Fe and S levels in their forage-based diet. This trial used twice-daily feeding, which has been proven to improve rumen pH and mineral solubility when compared to once-daily feeding or *ad libitum* systems. Abdelrahman and Hunaiti (55) utilized a comparable feeding frequency; however, Pal et al. (23) used a more flexible feeding schedule, which may explain some of the discrepancies in mineral dynamics. These findings suggest that the intermediate dose (YS1.50) was more successful than the higher dose (YS3.00) in increasing systemic mineral availability. The superior response at 1.50 g/day compared to 3.00 g/day may reflect microbial saturation at higher yeast inclusion levels or competition among minerals for shared transport pathways, as suggested by Abdelrahman and Hunaiti (55) and Zhang et al. (50). In this study, linear and quadratic contrasts revealed significant dose trends for several serum and rumen trace minerals, confirming a dose-dependent response to yeast supplementation. Abdelrahman and Hunaiti (55) also found that modest yeast inclusion resulted in excellent results, indicating a non-linear dose–response relationship, most likely due to microbial saturation or competing mineral absorption mechanisms. Zhang et al. (50) confirmed the superiority of Se yeast over inorganic Se for tissue deposition. This is consistent with the organic form’s better bioavailability.

The lack of individual TM intake measurements between the groups may restrict the interpretation of the results, and the study recommends that future studies incorporate individual intake monitoring for more precise assessment. Overall, these comparisons reveal that the response of trace mineral metabolism to yeast supplementation is complex, influenced by baseline diet composition, mineral type and dose, feeding frequency, rumen microbial ecology, and environmental factors. The obtained data support the idea that appropriate yeast inclusion rates (1.50 g/day) can increase the systemic availability of important trace minerals while remaining within tolerable limits, thereby boosting nutrient utilization efficiency in growing lambs.

## 5 Conclusion

Dietary yeast supplementation at a level of 1.50 mg/kg led to a numerical increase in feed consumption and weight gain in the growing lambs. More importantly, yeast culture supplementation demonstrated beneficial effects on trace mineral metabolism across rumen fluid, blood serum, and meat samples, with positive correlations observed between these tissues. These improvements occurred without any adverse effects on lamb growth performance or welfare. TM concentrations were significantly elevated in blood serum, reduced in rumen fluid, and enhanced in meat tissue. Although some minerals exhibited potential synergistic or antagonistic interactions, these effects were not statistically significant. Further research is recommended to better understand the complex dynamics and interactions of TM in response to dietary yeast supplementation.

## Data Availability

The original contributions presented in the study are included in the article/supplementary material, further inquiries can be directed to the corresponding authors.

## References

[1] Zheng S Li Y Chen C Wang N Yang F. Solutions to the dilemma of antibiotics use in livestock and poultry farming: regulation policy and alternatives. Toxics. (2025) 13:348. doi: 10.3390/toxics13050348, PMID: 40423429 PMC12115607

[2] Singh AS Devi MS Nongthomba U. Exploring global antibiotic use: a focus on seafood In: Antibiotic residue and resistance in seafood safety and quality. India: Springer. Ed. Upendra Nongthomba (2025). 1–26.

[3] Oladeji OM Mugivhisa LL Olowoyo JO. Antibiotic residues in animal products from some African countries and their possible impact on human health. Antibiotics. (2025) 14:90. doi: 10.3390/antibiotics14010090, PMID: 39858375 PMC11759178

[4] Gorzelanna Z Mamrot A Będkowska D Bubak J Miszczak M. Exploring the potential of novel animal-origin probiotics as key players in one health: opportunities and challenges. Int J Mol Sci. (2025) 26:5143. doi: 10.3390/ijms26115143, PMID: 40507953 PMC12154059

[5] Bozakova N Ivanov V. Possibilities of using *Saccharomyces cerevisiae* as a dietary supplement in sheep production. J Hyg Eng Design. (2023) 5, 11:43.

[6] Cuenca M Chauca J García C Sigüencia H. *Saccharomyces cerevisiae* as a replacement alternative to growth-promoting antibiotics in animal feed. Archivos de zootecnia. (2022) 71:61–9.

[7] Wang J Zhao G Zhuang Y Chai J Zhang N. Yeast (*Saccharomyces cerevisiae*) culture promotes the performance of fattening sheep by enhancing nutrients digestibility and rumen development. Fermentation. (2022) 8:719. doi: 10.3390/fermentation8120719

[8] Zhang Q Ma L Zhang X Jia H Tana GY Zhang J. Feeding live yeast (*Saccharomyces cerevisiae*) improved performance of mid-lactation dairy cows by altering ruminal bacterial communities and functions of serum antioxidation and immune responses. BMC Vet Res. (2024) 245:1–12. doi: 10.1186/s12917-024-04073-0, PMID: 38849835 PMC11157803

[9] Adam I Aruwayo A Garba M. Effect of feeding ensiled groundnut (*Arachis hypogaea*) shell with yeast (*Saccharomyces cerevisiae*) and molasses on performance and nutrient digestibility of Yankasa rams. FUDMA J Anim Prod Environ Sci Pollut Res. (2025) 1:28–36. doi: 10.33003/japes.2025.v1i1.28-36

[10] Xu J Li X Fan Q Zhao S Jiao T. Effects of yeast culture on lamb growth performance, rumen microbiota, and metabolites. Animals. (2025) 15:738. doi: 10.3390/ani15050738, PMID: 40076021 PMC11899153

[11] Obeidat BS Al-Khazaleh J Thomas MG Obeidat MD Nusairat BM. Dietary inclusion of olive cake alone or in combination with *Saccharomyces cerevisiae* in black goat kids: implications for performance and health. Veterinary World. (2024) 17:2497–505. doi: 10.14202/vetworld.2024.2497-2505, PMID: 39829653 PMC11736364

[12] Sun X Wang H You P Pacheco D Wang M Wu T. Agglomerated live yeast (*Saccharomyces cerevisiae*) supplemented to pelleted total mixed rations improves the growth performance of fattening lambs. Livest Sci. (2022) 258:104855. doi: 10.1016/j.livsci.2022.104855

[13] Zhang J Jin W Jiang Y Xie F Mao S. Response of milk performance, rumen and hindgut microbiome to dietary supplementation with aspergillus oryzae fermentation extracts in dairy cows. Curr Microbiol. (2022) 79:113. doi: 10.1007/s00284-022-02790-z, PMID: 35184209

[14] Xue L Zhou S Wang D Zhang F Li J Cai L. The low dose of *Saccharomyces cerevisiae* is beneficial for rumen fermentation (both in vivo and in vitro) and the growth performance of heat-stressed goats. Microorganisms. (2022) 10:1877. doi: 10.3390/microorganisms10101877, PMID: 36296154 PMC9609204

[15] Bohman W Nokkeaw T Chujai S Kotcharat J Daorueng R Sriboonnak R. The effects of dry yeast supplementation on rumen ciliated Protozoa population and volatile fatty acid production in lactating beef cows. Recent Sci Technol. (2025) 17:261372.

[16] Marri F Rehman A Awan GM Ahmad M Hassan M Ali C. Effect of yeast as prebiotics in small and large ruminants diet. Res Med Sci Rev. (2024) 2:87–99.

[17] Phesatcha K Phesatcha B Chunwijitra K Wanapat M Cherdthong A. Changed rumen fermentation, blood parameters, and microbial population in fattening steers receiving a high concentrate diet with *Saccharomyces cerevisiae* improve growth performance. Vet Sci. (2021) 8:294. doi: 10.3390/vetsci8120294, PMID: 34941821 PMC8707694

[18] Kulkarni NA Chethan H Srivastava R Gabbur AB. Role of probiotics in ruminant nutrition as natural modulators of health and productivity of animals in tropical countries: an overview. Trop Anim Health Prod. (2022) 54:110. doi: 10.1007/s11250-022-03112-y, PMID: 35195775

[19] Fróes R Bezerra L Castro D Barbosa A Arce-Cordero J Missasse J. Effects of yeast and exogenous fibrolytic enzyme inclusion in the diet of hair lambs on performance, carcass traits, physicochemical parameters and meat fatty acid profile. J Anim Feed Sci. (2024) 33:331–41. doi: 10.22358/jafs/176069/2024

[20] Jiang Y Dhungana A Odunfa OA McCoun M McGill J Yoon I. Effects of *Saccharomyces cerevisiae* fermentation product on ruminal fermentation, total tract digestibility, blood proinflammatory cytokines, and plasma metabolome of Holstein steers fed a high-grain diet. Transl Anim Sci. (2025) 9:txaf058. doi: 10.1093/tas/txaf058 40391287 PMC12086543

[21] Cole N Purdy C Hutcheson D. Influence of yeast culture on feeder calves and lambs. J Anim Sci. (1992) 70:1682–90. doi: 10.2527/1992.7061682x, PMID: 1634392

[22] Abdelrahman MM. Effect of direct-fed microbial (DFM)^®^ supplements on general performance of newborn awassi lambs. Egypt J Sheep Goats Sci. (2010) 5:1–18. doi: 10.5555/20113183126

[23] Pal K Paul SK Biswas P Patra AK Bhunia T Pakhira MC. Responses of addition of yeast (*Saccharomyces cerevisiae*) from rice distillers grains with solubles with or without trace minerals on the performance of black Bengal kids. Small Rumin Res. (2010) 94:45–52. doi: 10.1016/j.smallrumres.2010.06.006

[24] NRC. Nutrient requirements of small ruminants: Sheep, goats, cervids, and new world camelids. Washington. US: China Legal Publishing House (2007).

[25] Suttle N. Mineral nutrition of livestock. Mineral Nutrition of Livestock. 5th ed. Wallingford, UK: CABI: Cabi GB; (2022).

[26] Adeniji YA Sanni MO Abdoun KA Samara EM Al-Badwi MA Bahadi MA. Resilience of lambs to limited water availability without compromising their production performance. Animals. (2020) 10:1491. doi: 10.3390/ani10091491, PMID: 32846948 PMC7552272

[27] Harby AA Kholif AE Hamdon H. Assessment of *Saccharomyces cerevisiae* as a feed additive to improve the metabolic status and reproductive performance of Farafra ewes in arid subtropical regions. New Valley J Agric Sci. (2024) 4. doi: 10.21608/nvjas.2025.341880.1299

[28] Naseem C Rabbani I Rashid M Yousaf M Imran M Anjum A. Effects of the dietary supplementation of a native probiotic on the blood metabolites, gut parameters, and meat quality of growing lambs. S Afr J Anim Sci. (2024) 54:673–84. doi: 10.4314/sajas.v54i6.07

[29] Maamouri O Jemmali B Amrawi M Selmi H Rouissi H. Effects of yeast (*Saccharomyces cerevisiae*) feed concentrate supplement on growth performances and microbial activity in the rumen of “queue fine de L’ouest” lambs. J New Sci. (2016) 14:1297–302.

[30] Kewan K Ali M Ahmed B El-Kolty SA Nayel U. The effect of yeast (*saccharomyces cerevisae*), garlic (*allium sativum*) and their combination as feed additives in finishing diets on the performance, ruminal fermentation, and immune status of lambs. Egyp J Nutr Feeds. (2021) 24:55–76. doi: 10.21608/ejnf.2021.170304

[31] AOAC. Official methods of analysis of the Association of Analytical Chemists International Official Methods. Gaithersburg, MD, USA. AOAC International (2006).

[32] Azzam MM Qaid MM Al-Mufarrej SI Al-Garadi MA Albaadani HH Alhidary IA. Rumex nervosus leaves meal improves body weight gain, duodenal morphology, serum thyroid hormones, and cecal microflora of broiler chickens during the starter period. Poult Sci. (2020) 99:5572–81. doi: 10.1016/j.psj.2020.08.023, PMID: 33142474 PMC7647857

[33] Heyer CME Dörper A Sommerfeld V Gänzle MG Zijlstra RT. Effect of acidification or fermentation of barley grain using *Limosilactobacillus reuteri* or *Weissella cibaria* on inositol phosphate hydrolysis *in vitro*. Anim Feed Sci Technol. (2024) 309:115887. doi: 10.1016/j.anifeedsci.2024.115887

[34] Mokhtar MH Suliman AIA Abdou SG. Effect of macro and microalgae supplementation on productive performance, some blood constitutes and economic efficiency of growing Farafra male lambs. Arch Agric Sci J. (2023):73–83. doi: 10.21608/aasj.2023.295398

[35] Tripathi M Karim S. Effect of individual and mixed live yeast culture feeding on growth performance, nutrient utilization and microbial crude protein synthesis in lambs. Anim Feed Sci Technol. (2010) 155:163–71. doi: 10.1016/j.anifeedsci.2009.11.007

[36] Malekkhahi M Tahmasbi AM Naserian AA Danesh Mesgaran M Kleen J Parand A. Effects of essential oils, yeast culture and malate on rumen fermentation, blood metabolites, growth performance and nutrient digestibility of Baluchi lambs fed high-concentrate diets. J Anim Physiol Anim Nutr. (2015) 99:221–9. doi: 10.1111/jpn.12230, PMID: 25060172

[37] He T Mahfuz S Piao X Wu D Wang W Yan H. Effects of live yeast (*Saccharomyces cerevisiae*) as a substitute to antibiotic on growth performance, immune function, serum biochemical parameters and intestinal morphology of broilers. J Appl Anim Res. (2021) 49:15–22. doi: 10.1080/09712119.2021.1876705

[38] Saleem A Zanouny A Singer A. Growth performance, nutrients digestibility, and blood metabolites of lambs fed diets supplemented with probiotics during pre-and post-weaning period. Asian Australas J Anim Sci. (2016) 30:523–30. doi: 10.5713/ajas.16.0691, PMID: 28002935 PMC5394838

[39] Elaref M Hamdon H Nayel U Salem A Anele U. Influence of dietary supplementation of yeast on milk composition and lactation curve behavior of Sohagi ewes, and the growth performance of their newborn lambs. Small Rumin Res. (2020) 191:106176. doi: 10.1016/j.smallrumres.2020.106176

[40] Rios CAV. Effect of source of trace minerals on nutrient digestibility and rumen fermentation of dairy cows South Carolina: Clemson University (2024).

[41] Fadlalla IM. The interactions of some minerals elements in health and reproductive performance of dairy cows In: New advances in the dairy industry (2022). Ed. Muhammad Subhan Qureshi. IntechOpen: University of Agriculture, Pakistan.

[42] Waghorn G Clark D. Feeding value of pastures for ruminants. N Z Vet J. (2004) 52:320–31. doi: 10.1080/00480169.2004.36448, PMID: 15768132

[43] Dmytruk O Yemets A Dmytruk K. Yeasts as biofertilizers and biocontrol agents: mechanisms and applications. Biotechnol Appl Biochem. (2025) doi: 10.1002/bab.70029 40643311

[44] Sadr VS Nguyen HTT Pineda L Han Y Barekatain R Toghyani M. Hydroxychloride zinc and copper supplementation improves growth, feed efficiency, and gut health in broiler chickens. Sci Rep. (2025) 15:31903. doi: 10.1038/s41598-025-17713-8, PMID: 40883514 PMC12397401

[45] Saha SK Pathak NN Saha SK Pathak NN. Mineral nutrition In: Fundamentals of animal nutrition (2021). 113–31.

[46] McDowell L. Minerals in animal and human nutrition. (2003). San Diego. USA: Academic Press Inc.

[47] Lee J Knowles S Judson G. Trace-element and vitamin nutrition of grazing sheep In: Sheep nutrition. Ed. M. Freer. Wallingford UK: CABI Publishing (2002). 285–311.

[48] Zhang W Zhang Y Zhang SW Song XZ Jia ZH Wang RL. Effect of different levels of copper and molybdenum supplements on serum lipid profiles and antioxidant status in cashmere goats. Biol Trace Elem Res. (2012) 148:309–15. doi: 10.1007/s12011-012-9380-2, PMID: 22407467

[49] Kumar R Sahu DS Chandra G Yadav SP Kumar R Ali N. Effect of Astaxanthin and copper supplementation on growth, immunity, antioxidant, and blood biochemical status of growing murrah buffalo heifers. Biol Trace Elem Res. (2022) 200:5052–63. doi: 10.1007/s12011-021-03091-5, PMID: 35061144

[50] Zhang S Wu Z Heng J Song H Tian M Chen F. Combined yeast culture and organic selenium supplementation during late gestation and lactation improve preweaning piglet performance by enhancing the antioxidant capacity and milk content in nutrient-restricted sows. Anim Nutr. (2020) 6:160-167. doi: 10.1016/j.aninu.2020.01.004, PMID: 32542196 PMC7283508

[51] Klein GS Leal KW Rodrigues CA Draszevski TM Brunetto AL Vitt MG. Organic zinc and selenium supplementation of late lactation dairy cows: effects on milk and serum minerals bioavailability, animal health and milk quality. Animals. (2025) 15:499. doi: 10.3390/ani15040499, PMID: 40002983 PMC11852322

[52] Gowda N Pal D Dey DK. Organic trace minerals in ruminant nutrition: production, reproduction, health, economics, and environmental implications In: Ed. Mahesh, M.S., Yata, V.K. Feed additives and supplements for ruminants. Singapore: Springer (2024). 69–86.

[53] Abdelrahman MM Alhidary IA Matar AM Alobre MM Alharthi AS Faye B. Effect of total mixed ratio (TMR) supplementation on milk nutritive value and mineral status of female camels and their calves (*Camelus dromedarius*) raised under semi intensive system during winter. Agriculture. (2022) 12:1855. doi: 10.3390/agriculture12111855

[54] McDowell LR. Minerals in animal and human nutrition. San Diego. USA: Academic Press Inc. (1992).

[55] Abdelrahman MM Hunaiti DA. The effect of dietary yeast and protected methionine on performance and trace minerals status of growing Awassi lambs. Livest Sci. (2008) 115:235–41. doi: 10.1016/j.livsci.2007.07.015

[56] Demir AÖ Karakuş F Akkol S. An investigation on serum mineral levels of healthy Norduz and hair goats raised in semi-intensive conditions. Turk J Agric. (2020) 8:1795–802. doi: 10.24925/turjaf.v8i8.1795-1802.3520

[57] Ciliberti M Caroprese M Albenzio M. Climate resilience in small ruminant and immune system: an old alliance in the new sustainability context. Small Rumin Res. (2022) 210:106662. doi: 10.1016/j.smallrumres.2022.106662

[58] Zou J Wei L Liang Y Zou J Cheng P Mo Z. Impact of heat stress on gene expression in the hypothalamic–pituitary–ovarian Axis of Hu sheep. Animals. (2025) 15:2189. doi: 10.3390/ani15152189, PMID: 40804979 PMC12345516

[59] Arthington JD Ranches J. Trace mineral nutrition of grazing beef cattle. Animals. (2021) 11:2767. doi: 10.3390/ani11102767, PMID: 34679788 PMC8532955

